# Circulating small extracellular vesicles increase after an acute bout of moderate-intensity exercise in pregnant compared to non-pregnant women

**DOI:** 10.1038/s41598-021-92180-5

**Published:** 2021-06-16

**Authors:** Shuhiba Mohammad, Kelly Ann Hutchinson, Danilo Fernandes da Silva, Jayonta Bhattacharjee, Kurt McInnis, Dylan Burger, Kristi B. Adamo

**Affiliations:** 1 Faculty of Health Sciences, School of Human Kinetics, University of Ottawa, Ottawa, ON Canada; 2 Kidney Research Centre, Department of Cellular and Molecular Medicine, The Ottawa Hospital Research Institute, University of Ottawa, Ottawa, ON Canada; 3 200 Lees Avenue, Building E, Room E250F, Ottawa, ON K1N 6N5 Canada

**Keywords:** Reproductive biology, Physiology

## Abstract

The physiological and molecular mechanisms linking prenatal physical activity and improvements in maternal–fetal health are unknown. It is hypothesized that small extracellular vesicles (EVs, ~ 10–120 nm) are involved in tissue cross-talk during exercise. We aimed to characterize the circulating small EV profile of pregnant versus non-pregnant women after an acute bout of moderate-intensity exercise. Pregnant (N = 10) and non-pregnant control (N = 9) women performed a single session of moderate-intensity treadmill walking for 30 min. Plasma was collected immediately pre- and post-exercise, and small EVs were isolated by differential ultracentrifugation. EV presence was confirmed by western blotting for the small EV proteins TSG-101 and flottilin-1. Small EVs were quantified by size and concentration using nanoparticle tracking analysis and transmission electron microscopy. All EV fractions were positive for TSG-101 and flotillin-1, and negative for calnexin. Mean vesicle size at baseline and percent change in size post-exercise were not different between groups. At baseline, pregnant women had higher levels of small EVs compared to controls (1.83E+10 ± 1.25E+10 particles/mL vs. 8.11E+09 ± 4.04E+09 particles/mL, respectively; *p* = 0.032). Post-exercise, small EVs increased significantly in the circulation of pregnant compared to non-pregnant women after correcting for baseline values (64.7 ± 24.6% vs. − 23.3 ± 26.1%, respectively; F = 5.305, *p* = 0.035). Further research is needed to assess the functional roles of exercise-induced small EVs in pregnancy.

## Introduction

Engagement in regular physical activity is associated with significant health benefits, including improvements in both psychological and physiological well-being and decreased risk of chronic disease and all-cause mortality^1^. Physical activity is also an important component of a healthy pregnancy. The *2019 Guidelines for Physical Activity Throughout Pregnancy* recommend that women without contraindication to exercise, engage in a minimum of 150 min of moderate-intensity physical activity per week, with engagement every day being preferable^2^. Physical activity during pregnancy is associated with both short- and long-term benefits for the mother and fetus, which include lowering the risk for adverse pregnancy complications such as gestational diabetes mellitus, preeclampsia, gestational hypertension, excessive gestational weight gain, and macrosomia (large for gestational-age neonates)^3–6^. Although the benefits of chronic prenatal physical activity are extensive and well-known, the physiological mechanisms involved in response to exercise in pregnancy leading to these benefits remain largely unclear. The benefits sustained from engagement in chronic exercise are derived from acute physiological changes promoted by a single bout of exercise experienced over time^7,8^. During exercise, several molecules are released into circulation, including catecholamines^9,10^, myokines^11,12^, and small extracellular vesicles (EVs)^7,13^, which seem to mediate the adaptations caused by the cumulative effect of daily bouts of ‘acute’ physical activity (i.e., the chronic effects of exercise)^14^.

In the non-pregnant population, small EVs (20–120 nm in diameter, often referred to as exosomes) are one of the potential mechanisms proposed to modulate the systemic benefits of exercise^7,14,15^. EVs are a heterogeneous group of lipid bilayered membrane nanoparticles secreted by cells into extracellular space^16,17^. EVs are believed to be involved in intercellular communication over long distances as they may facilitate the transfer or interaction of biologically active molecules, including nucleic acids (mRNAs and other small RNAs, i.e., microRNAs), proteins, and lipids from donor cells to target cells^18,19^. EVs are found in a wide range of biological fluids, including blood, urine, saliva, milk, and cells in culture^20^. Their involvement in a multitude of physiological and pathophysiological processes has led to their emergence as particles of interest in the complex realm of the physiological response to exercise.

Work from our lab has shown that the acute physiological response to exercise, relating to the release of soluble factors like cytokines into circulation, is different in pregnant compared to non-pregnant women^21^. It remains unknown whether acute prenatal exercise results in a change in the levels of circulating small EVs, and whether the response differs depending on pregnancy status. It is known that plasma concentrations of small EVs are significantly higher in pregnant versus non-pregnant women^22^. It is thus posited that the major contributor accounting for this difference is the placenta, the critical organ of pregnancy^22,23^. Evidence supports the beneficial impact of both acute and chronic prenatal exercise on the placenta^21,24–30^; however, the mechanisms are currently unknown. Small EVs are involved in maternal–fetal communication^23,31^, and therefore, warrant further investigation in the context of exercise during pregnancy.

In males, small EVs are released rapidly into circulation after an acute bout of sustained, vigorous-intensity exercise^7,13^, and are thought to be involved in mediating intercellular communication that can contribute to the chronic adaptations of exercise^7^. However, there are no studies on the impact of acute exercise on the levels of small EVs in the circulation of pregnant or non-pregnant women. We aimed to characterize the plasma small EV profile and concentration after a single bout guidelines-based of moderate-intensity exercise in pregnant women, compared to their non-pregnant counterparts. To our knowledge, this is the first study to examine small EVs in response to acute exercise in pregnant and non-pregnant women.

## Methods

### Ethical approval

All experimental procedures were approved by the University of Ottawa Research Ethics Board (file number: H-06-18-634) and conducted following the ethical principles outlined in the Declaration of Helsinki. Informed written consent was obtained from all study participants after an explanation of study procedures and objectives.

### Study participants

Pregnant and non-pregnant women were recruited from the Ottawa region (Ontario, Canada), and eligibility was assessed by the research team. Healthy women between the ages of 18–40 years with no contraindications to exercise were included. Participants were weight-stable (± 5 kg) for at least six months before the study, with a self-reported pre/non-pregnant body mass index (BMI) of 18.5–29.9 kg/m^2^. Those with untreated thyroid disease, hypertension, diabetes (pre/non-pregnant or gestational diabetes), or other chronic health conditions, and frequent users of drugs, tobacco, or alcohol were excluded. Moreover, pregnant participants needed to be between 13 and 28 weeks of gestation and carrying a singleton fetus to be included. In total, 10 pregnant and 9 non-pregnant women met inclusion criteria and were included in this study.

### Experimental procedure

The experimental protocol followed methodologies outlined by Hutchinson et al*.* (2019)^21^. Briefly, study participants were asked to abstain from exercise for 12 h and food for 8 h before the experimental session. Body weight and height were measured using standard methods. Participants were provided with a standardized snack of approximately 340 kCal before the exercise session. Then, resting heart rate (HR) was measured using a Polar V800 HR monitor (Polar Electro, Lachine, QC) for 10 min while participants were comfortably seated. The values obtained during the last 5 min of measurement (recorded at 1-min intervals) were averaged to determine resting HR. A target of 40–59% of HR reserve (HRR) was used to specify moderate-intensity exercise^2,32^ and calculated using the following equation^33^:

$$\begin{aligned} & {\text{Target}}\,{\text{HR}}\,{\text{range}}\, = \,\left[ {\left( {{\text{Maximal}}\,{\text{HR}}\,{-}\,{\text{resting}}\,{\text{HR}}} \right)*\,\% {\text{intensity}}} \right] + {\text{resting}}\,{\text{HR}}. \\ & {\text{Maximal}}\,{\text{HR}} = 220{\text{-age}} \\ \end{aligned}$$

A 3-min warm-up at 2.0 miles per hour (mph) at an incline of 2.0% was followed by an increase to 6.0% incline, where the speed increased by 0.2 mph at 1-min intervals until the upper range of the target moderate-intensity HR was reached (approaching 59% HRR). Once this range was met, the participants continued to exercise for 30 min, adjusting the speed accordingly to ensure that the target HR range was maintained. HR values were recorded at 1-min intervals during the 30 min of continuous moderate-intensity exercise. Approximately 14–20 min after the snack, immediately before and after the exercise session, 10 mL of blood was drawn from the median cubital vein using a BD Vacutainer® Safety-Lok™ blood collection set (#367281; BD Biosciences, Mississauga, ON) and potassium EDTA (#367863; BD Biosciences) collection tubes. To obtain plasma, whole blood was immediately centrifuged at 1700×*g* for 15 min at 4 °C. Plasma samples were stored at − 80 °C until further analysis.

### Small EV isolation

Isolation of small EVs was achieved by differential ultracentrifugation as previously described^34,35^. Frozen plasma samples (1.0 mL) were thawed at 37 °C. After thawing, plasma samples were always kept on ice. Samples were centrifuged at 20,000 × *g* for 20 min at 4 °C to remove apoptotic bodies and large EVs. The resulting supernatant was centrifuged at 100,000×*g* using a Beckman Coulter Optima MAX ultracentrifuge (Beckman Coulter Inc., Brea, CA, USA) with a TLA-55 rotor (Beckman Coulter) for 90 min at 4 °C. The resulting small EV pellet was washed with 1.0 mL of 0.1 µm-filtered phosphate-buffered saline (PBS) and then centrifuged at 100,000×*g* as described above. The final small EV pellet was resuspended in 100 µL of 0.1 µm-filtered PBS. Aliquots of 10 µL were separated and frozen at − 80 °C for further analysis.

### Western blot analysis

Western blotting was performed as described by Bhattacharjee et al*.* (2010)^36^, with some modifications, to test for the presence and absence of small EV markers. Immediately following isolation, 1 µL of 10X radioimmunoprecipitation assay (RIPA) buffer with protease inhibitor cocktail (#P8340, MilliporeSigma Canada Co, Oakville, ON, Canada) was added to 10 µL of small EV isolates in PBS. Samples were then sonicated for 1 min to achieve protein lysis. An equal volume of protein lysates (5 µL) from each sample was subjected to SDS-PAGE electrophoresis under reducing conditions using a 4–15% mini-protean TGX precast gel (Bio-Rad Laboratories, Mississauga, ON, Canada) at 120 V for 60 min. Separated proteins were blotted onto a PVDF membrane (Bio-Rad Laboratories) at 100 V for 90 min. Blots were then blocked in 5% non-fat dry milk in TBS with 0.1% TWEEN®20 (TBST) for 60 min at room temperature. After blocking, blots were incubated with primary antibodies for markers of small EVs, flotillin-1 (mouse anti-flotillin-1, 1:1000; #610820; BD Biosciences, CA, USA) and tumor susceptibility gene 101 (TSG101) (mouse anti-TSG101, 1:3000; #ab83; Abcam Inc, Toronto, ON, Canada), and for the negative control, calnexin (rabbit anti-calnexin, 1:10,000; #AB2301; Millipore Sigma), in 5% milk in TBST overnight at 4 °C. Following overnight incubation, the blots were washed three times with TBST and incubated with goat anti-mouse (1:7000; #1706516; Bio-Rad Laboratories) or goat anti-rabbit secondary antibody coupled with HRP (1:7000; #1706515; Bio-Rad Laboratories) diluted in 5% milk in TBST for 60 min at room temperature. After washing three times with TBST, the peroxidase-labeled blots were incubated with Clarity Western ECL Substrate (#1705060; Bio-Rad Laboratories) and visualized using a ChemiDoc XRS + system (Bio-Rad Laboratories). Protein lysates from BeWo choriocarcinoma cells and term human placenta tissue were used as positive controls for the calnexin immunoblots.

### Nanoparticle tracking analysis

Particle size and concentration were determined using nanoparticle tracking analysis (NTA) as described previously^35,37^. Dilutions of small EV isolates (in 0.1 µm-filtered PBS ranging from 1:250 to 1:1000) were analyzed using the ZetaView PMX 110 Multiple Parameter Particle Tracking Analyzer (Particle Metrix, Meerbusch, Germany) in size mode with Zetaview software (version 8.03.08). Small EVs were captured at 11 camera positions at 21 °C with the following pre-acquisition parameters: shutter speed of 70, 30 frames per second. The following post-acquisition settings were used: minimum brightness of 30, minimum tracelength of 15, minimum size of 20, and maximum size of 1000.

### Transmission electron microscopy

Small EVs were examined by transmission electron microscopy (TEM) as previously described^38^. Briefly, small EVs were pelleted by ultracentrifugation at 100,000×*g* for 90 min at 20 °C. The supernatant was aspirated, and the small EV pellet was fixed with 2.5% glutaraldehyde in PBS for four hours at room temperature. The pellet was then washed in 0.1 M Na cacodylate buffer, post-fixed in 2% OsO4, and dehydrated in a series of graded ethanols. Samples were then embedded in Spurr Resin, and 60 nm sections were prepared on copper grids. Samples were visualized using a JEOL JEM-1400 Plus electron microscope.

### Statistical analysis

All statistical analyses were conducted using SPSS version 23.0 (IBM Corp, Armonk, NY, USA), and graphs were created using GraphPad Prism version 8.4.2 (GraphPad Software, La Jolla, CA, USA). All data are shown as mean ± standard deviation. Normality was assessed using the Shapiro–Wilk test. Study participant demographics and baseline (pre-exercise) characteristics (size and concentration) of small EVs were compared between pregnant and non-pregnant women using independent *t*-tests. The change in HR during the 30 min continuous exercise session was assessed using a two-way mixed ANOVA with Bonferroni’s multiple comparisons post-test. The change in small EV size and concentration post-exercise was calculated using the following equation:

$${\text{Change}}\,{\text{in}}\,{\text{small}}\,{\text{EV}}\,{\text{size}}\,{\text{or}}\,{\text{concentration}}\, = \,\left( {{\text{post-exercise}}\,{-}\,{\text{pre-exercise}}} \right)/{\text{pre-exercise}}*100$$

An independent *t*-test was used to compare the change in small EV size in pregnant versus non-pregnant women. Due to the differences observed in the baseline levels of small EVs, an analysis of covariance (ANCOVA) with Bonferroni correction for post hoc comparisons was used to compare the change in small EV concentration, controlling for baseline values, in pregnant versus non-pregnant women. Adjusted means and standard errors are presented where applicable. Statistical significance was considered when *p* < 0.05.

### Ethics approval

This study was performed in accordance with all aspects of the Declaration of Helsinki. The Research Ethics Board of the University of Ottawa granted full ethical approval (File number: H11-15-29). All participants conferred informed written consent after explanation and review of all study protocols and procedures.

## Results

### Study participant demographics

**Figure 1:**
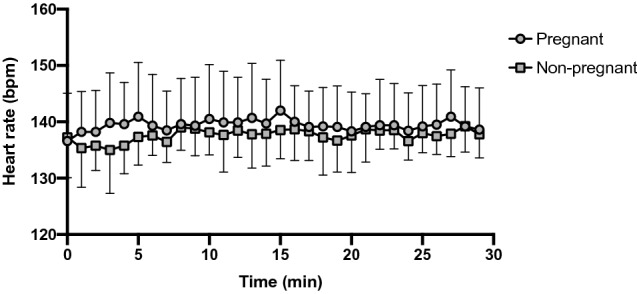
Heart rate during the continuous moderate-intensity exercise session. The change in heart rate was not different between pregnant and non-pregnant women across the 30 min moderate-intensity exercise session**.** The main effects of time and pregnancy were not significantly different (F = 1.1, *p* = 0.38 and F = 0.50, *p* = 0.50, respectively), nor was the time × pregnancy interaction (F = 0.55, *p* = 0.97). Data are shown as mean ± standard deviation. Bpm, beats per minute.

All study participant demographics are presented in Table 1. By design, pregnant and non-pregnant women did not differ in anthropometric variables, including age, height, pre/non-pregnant weight, pre/non-pregnant BMI and resting HR. Also, the time to reach the moderate intensity HR range (approaching 59% HRR) was not different between groups, nor was the total exercise duration (Table 1). Further, the average speed achieved and the change in HR during the moderate-intensity exercise session were not different between groups (Table 1 and Fig. 1).

**Table 1:** Study participant demographics.

	Pregnant (N = 10)	Non-pregnant (N = 9)	*p* value
Age (years)	31.1 ± 2.7	32.7 ± 3.2	0.68
Gestational age (weeks)	20.6 ± 5.4	–	–
Height (cm)	167.0 ± 5.3	163.8 ± 7.2	0.28
Pre/non-pregnant weight (kg)	62.7 ± 8.6	59.8 ± 8.6	0.50
Pre/non-pregnant BMI (kg/m^2^)	23.4 ± 3.3	22.3 ± 2.8	0.43
Resting heart rate (bpm)	77.9 ± 10.9	69.2 ± 6.6	0.052
Time to reach moderate intensity heart rate range (min)	10.4 ± 2.2	11.8 ± 1.9	0.16
Heart rate during continuous exercise session (bpm)	139.4 ± 7.5	137.6 ± 5.0	0.55
Average speed during continuous exercise session (mph)	3.4 ± 0.5	3.6 ± 0.4	0.18
Total exercise duration (min)	40.4 ± 2.2	41.8 ± 1.9	0.16

Data are presented as mean ± standard deviation. BMI, body mass index; bpm, beats per minute; mph, miles per hour; SD, standard deviation. Data were analyzed using independent *t*-tests.

### Small EV isolates exhibit classical characteristics

**Figure 2:**
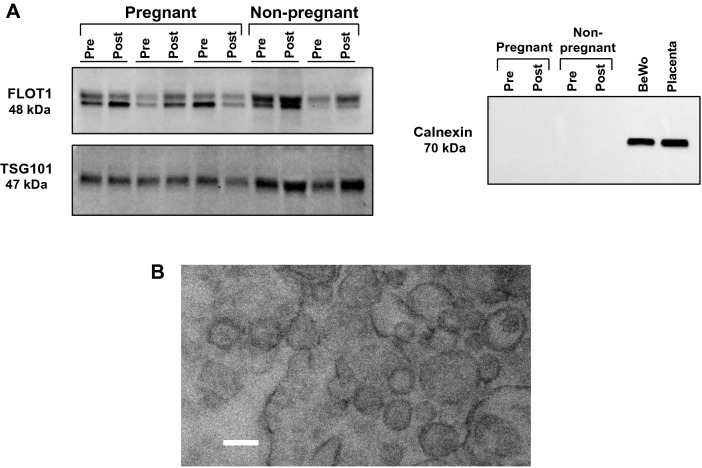
Circulating small EV isolates both pregnant and non-pregnant women expressed markers of small EVs both pre- and post-exercise. (**A**) Cropped representative western blot images show the positive expression of flotillin-1 (FLOT1) and tumor suppressor gene-101 (TSG101) in small EV isolates from pregnant and non-pregnant women, both pre- (‘Pre’) and post-exercise (‘Post’). Calnexin, a negative control marker for small EVs, was not detected in small EV isolates from either group. Positive controls for calnexin expression included BeWo choriocarcinoma cell lysates (‘BeWo’) and term human placenta tissue lysates (‘placenta’). Each lane represents a sample from a single individual, while each pair of lanes were obtained from the same participant. Full length blots are presented in Supplementary Fig. 1. (**B**) Shows a representative transmission electron microscopy image of small EV pellets where round particles (~ 100 nm in size) with distinct, intact membranes can be seen (scale bar = 100 nm). EVs, extracellular vesicles.

Western blotting was used to qualitatively assess the presence of small EV protein markers, flotillin-1, and TSG101. Positive signals for flotillin-1 and TSG101 were found in protein lysates from small EV isolates of pregnant and non-pregnant women, both pre- and post-exercise (Fig. 2A). Calnexin, a calcium-binding protein found in the membrane of endoplasmic reticulum, was absent in small EV isolate fractions but positive in BeWo choriocarcinoma and human term placenta tissue lysates (Fig. 2A). Further, TEM analysis showed the presence of small particles (~ 100 nm in size) with distinct, intact membranes (Fig. 2B). Together, these data confirmed the presence of small EVs in the plasma samples of our study participants.

### Small EV isolates did not differ by size

**Figure 3:**
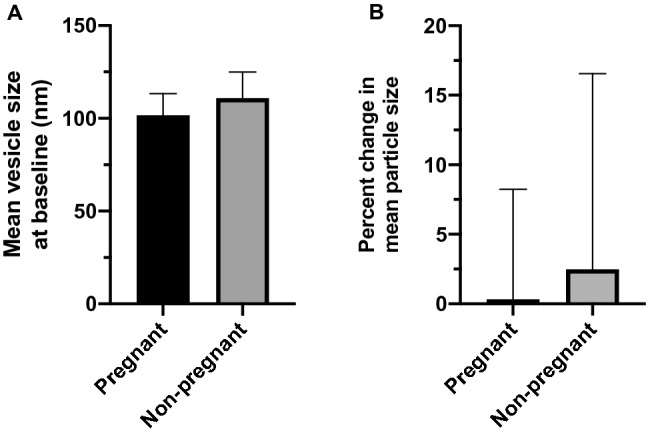
Small EVs did not differ in size at baseline or post-exercise in pregnant versus non-pregnant women. Using nanoparticle tracking analysis the mean size of circulating small EVs were not different at baseline (pre-exercise) in pregnant compared to non-pregnant women (*p* = 0.141) (**A**). Furthermore, the change in small EV size post-exercise was not different between groups (*p* = 0.683) (**B**). Data are shown as mean ± standard deviation. EVs, extracellular vesicles.

**Figure 4:**
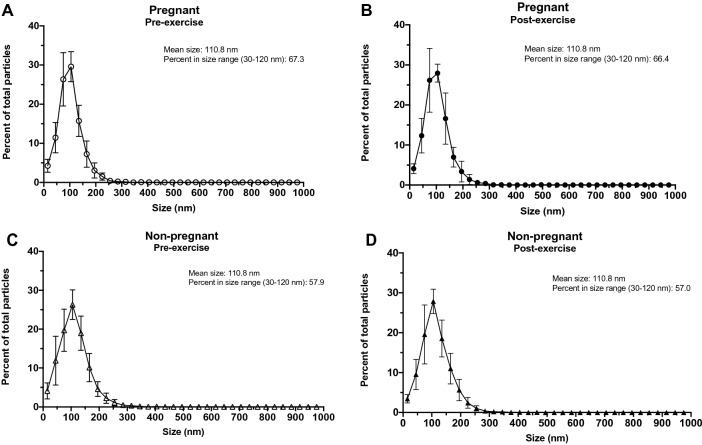
Size distribution of small EV isolates from pregnant and non-pregnant women both pre- and post-exercise. The size distribution of small EV isolates obtained from pregnant (**A**, **B**) and non-pregnant (C&D) obtained via nanoparticle tracking analysis are shown. All data points are displayed as mean ± standard deviation. EVs, extracellular vesicles.

Characterization of the mean vesicle size of small EV isolates revealed no differences in the baseline (pre-exercise) plasma samples of pregnant versus non-pregnant women (101.7 ± 11.7 nm and 110.8 ± 14.1 nm, respectively; *p* = 0.141) (Fig. 3A). There was no significant difference in vesicle size post-exercise in either pregnant or non-pregnant individuals (percent change of pregnant = 0.33 ± 7.9% and non-pregnant = 2.48 ± 14.1%; *p* = 0.683) (Fig. 3B). The size distributions of small EV isolates and the percentage of particles from 30–120 nm are shown in Fig. 4.

### Pregnant women display higher levels of small EVs before and after exercise compared to non-pregnant controls

**Figure 5:**
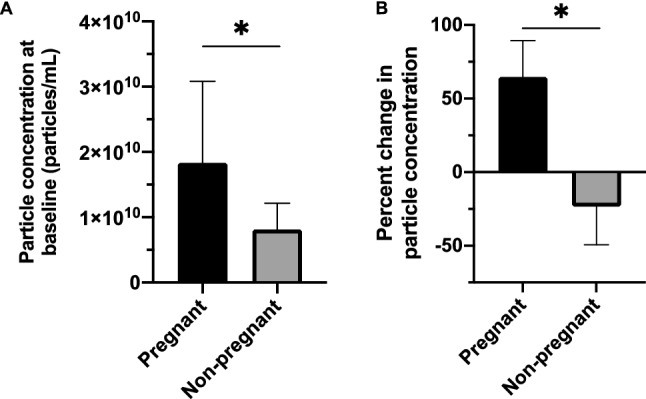
Pregnant women had higher levels of circulating small EVs at baseline and post-exercise compared to non-pregnant women. Nanoparticle tracking analysis revealed that pregnant women had higher levels of small EVs in plasma at baseline (pre-exercise) (*p* = 0.032) (**A**). (**B**) Furthermore, when accounting for baseline levels of small EVs, pregnant women were found to have significantly higher levels on average of small EVs post-exercise compared to non-pregnant women (F = 5.305, *p* = 0.035). Data are presented as mean ± standard deviation. *p < 0.05. EVs, extracellular vesicles.

First, the baseline (pre-exercise/rest) levels of small EVs were compared between pregnant and non-pregnant women. At rest, pregnant women had higher levels of small EVs in circulation compared to their non-pregnant counterparts (1.83E+10 ± 1.25E+10 particles/mL and 8.11E+09 ± 4.04E+09 particles/mL, respectively; *p* = 0.032) (Fig. 5A). When correcting for differences in baseline levels, small EVs increased significantly in the circulation of pregnant versus non-pregnant women post-exercise (adjusted means ± standard error: 64.7 ± 24.6% vs. − 23.3 ± 26.1% for pregnant and non-pregnant women, respectively; F = 5.305, *p* = 0.035)(Fig. 5B).

## Discussion

To the best of our knowledge, this is the first report characterizing small EVs in response to exercise in pregnant and non-pregnant women. We compared the circulating small EV profile of pregnant compared to age- and pre-pregnant BMI-matched non-pregnant women after an acute bout of moderate-intensity treadmill exercise. Small EV isolates from the plasma of pregnant and non-pregnant women expressed protein markers indicative of small EVs before and after an acute bout of exercise. The results also indicate that while there is no change in the size of small EVs in circulation post-exercise in healthy pregnant and non-pregnant women, pregnant women had significantly higher levels of small EVs in plasma after exercise.

Previous studies involving male subjects reported a rapid release of small EVs into circulation after sustained, high-intensity cycling, or running exercise until physical exhaustion^7,13^. We only found a significant difference in the number of small EVs post-exercise when comparing between groups after controlling for the baseline differences between pregnant and non-pregnant women, not within groups (data not reported). There may be a few reasons for this discrepancy. Frühbeis et al*.* (2015) quantified small EVs by NTA in a small subset of participants (N = 2 per exercise modality) and, therefore, could not be used to compare with the results in our study. Furthermore, the reported increase in small EVs after exercise was based on data from the semi-quantitative analysis of protein markers by western blotting^13^, which was not used to quantify the absolute number of small EVs of our study. Also, Whitham et al*.* (2018) observed an increase in particles in the size range attributed to small EVs, but it is not clear how many samples were quantified by NTA (only a ‘subset of samples’ is described) or what statistical tests were employed, and if significance was observed. In a study assessing the plasma small EV profile after exercise in human patients deemed ‘at-risk’ for cardiometabolic disease, an increase in the number of EVs without a difference in size was observed^39^. Our findings support exercise studies in animals, where differences were detected in the number of small EVs only and not in size^40^. We believe that the discrepancy between our results and those of the current human literature may be due to differences in the study population and exercise intensity, duration, and mode. Participants studied by Frühbeis and colleagues (2015) and Whitham et al*.* (2018) exercised at higher intensities or durations than our study participants (moderate-intensity exercise). Since we studied pregnant women, we opted to use a physiologically-relevant exercise stimulus, guided by *2019 Guidelines for Physical Activity Throughout Pregnancy*^2^, to more appropriately examine the potential changes in the small EV profile after exercise.

Small EVs are theorized to contain biologically-active materials involved in long-distance intercellular communication throughout the body. Regarding exercise, quantitative proteomic analysis of small EV cargo showed that they contained myokines^7^, peptides produced by skeletal muscles and thought to mediate cross-talk between muscles and other tissues during exercise ^11,12^. Critical studies involving animals revealed that exercise-induced small EVs localized to the liver, possibly regulating metabolism in response to exercise^7^. Furthermore, it has been hypothesized that the systemic and long-term benefits of exercise are mediated in part by small EVs^14^. The potential bioactivity of exercise-induced small EVs is not fully understood, but were found to be cardioprotective in a cardio-ischemic reperfusion injury mouse model^39^. Although the aim of the current study was not to characterize the biological contents of small EVs in circulation after exercise, further research is warranted to determine the physiological impact of small EVs specifically in the context of exercise in pregnancy given that we observed remarkable differences depending on pregnancy status in a healthy population of women.

Our results show that the physiological response to acute exercise may be different in pregnant versus non-pregnant women. Likewise, we have recently demonstrated that myokine profiles were different in pregnant and non-pregnant women after a matched-intensity exercise bout^21^. It has been hypothesized that the release of small EVs is triggered by an acute stress response in the body prompted by exercise^13^. It is, therefore, conceivable that the physiological stress response to exercise is heightened during pregnancy, which may help to explain our findings. It is not known whether the increase in small EV number observed post-exercise in pregnant women was due to de novo secretion or decreased clearance/uptake when compared to their non-pregnant counterparts, and requires further study. Interestingly, we found that on average, levels of small EVs decreased in non-pregnant women post-exercise whereas the opposite result was observed in pregnant women. Small EVs have been shown to be cleared from circulation in-part by members of the innate immune system, namely, macrophages^41,42^. It is well known that immunity in pregnancy shifts from a Th-1 or innate immunity-biased profile (cell-mediated immunity/pro-inflammatory) to that of one that is Th-2 or adaptive immunity-biased (humoral immunity/anti-inflammatory) response to tolerate the semi-allogeneic fetus^43,44^. This may explain why we observed a decrease in the concentrations of EVs in non-pregnant women after exercise, when immunity is Th-1-biased and macrophages may be playing a greater role in the clearance of small EVs from circulation. However, it is currently unknown whether the clearance of small EVs is impacted by immune status during pregnancy.

The differing responses to exercise-induced small EVs depending on pregnancy status is not surprising given that healthy pregnant women displayed a 50-fold increase in the concentration of small EVs in plasma compared to non-pregnant women^22^. We confirmed the increase in small EVs in pregnant versus non-pregnant women but failed to observe the same dramatic differences. This lack of validation may be due to differences in sample collection, processing, and analysis, but we noted the same trend. Nevertheless, we postulate that the placenta may contribute to the overall increase in small EVs observed after acute exercise, but necessitates further assessment. The placenta is known to release small EVs vital for mediating maternal-fetal communication^31,45–47^, and it is hypothesized that the placenta releases the majority of EVs in circulation during pregnancy, namely, from trophoblast cells^22,23^. Some reports estimate that between 12 and 25% of small EVs in maternal circulation have placental origins^48^. It has also been proposed that oxygen tension is inversely correlated with the release of small EVs by various placental cell types^22,49–51^. This evidence could provide an explanation for the differences in small EV levels after exercise, as it has been hypothesized that the placenta may experience intermittent hypoxia in response to exercise^52–54^. Studies have linked small EVs released by varying placental cell types in promoting vascular smooth muscle cell migration^50,55^ as well as endothelial cell migration and vasculogenesis^51^, critical processes involved in promoting proper angiogenesis of the feto-placental vasculature. Exercise is known to improve placental perfusion and vascular surface volume^27,28,56^. Therefore, it is plausible that small EVs could provide a mechanistic contribution to help understand how the benefits of exercise during pregnancy are bestowed upon the mother and fetus. However, further research is needed to establish the functional role of exercise-induced small EVs in healthy pregnancies.

Our study is not without limitations. Although our sample number was equivalent to or greater than reported in previous work (ranging from N = 4–11 per group^7,13^), the sample size is still small. An a priori sample size calculation could not be performed due to differences in the study population, exercise, and quantitative techniques used by other studies published in this field (i.e., differing study populations, sustained vigorous-intensity exercise, quantification by semi-quantitative methodologies). Despite our small sample size, we believe our exercise protocol was well-controlled due to the application of a matched-intensity exercise bout guided by HRR. Unlike other studies involving acute exercise and small EVs, we did not evaluate differing exercise intensities/modes or collect samples after exercise recovery or at multiple timepoints. We believe that a strength of our study was the type, duration, and intensity of the exercise selected for examination. Since walking is the preferred exercise for pregnant women^57,58^, we surmise our study presents strong applicability and generalizability. It is unknown whether the small EV profile could be affected by volume, intensity or mode of exercise in pregnant women. It is known that exercise modality impacted small EV release and/or clearance in men^13^. Future studies should evaluate differing exercise durations, intensities, and types of exercise (i.e., aerobic versus resistance training, weight-bearing versus non-weight bearing exercise). Another strength was that our groups were matched for age and pre/non-pregnant BMI to reduce potential confounding factors allowing for more robust comparisons to be made between groups. Further, we used differential ultracentrifugation to isolate small EVs, a technique known to produce low, yet highly specific yields^59,60^, allowing for further analysis using proteomic research methodologies. We did not investigate the molecular contents, cellular origin, or bioactivity of exercise-induced small EVs, a limitation of our current study, but is the subject of ongoing studies in this population. As of yet, the role of small EVs induced by acute exercise on the chronic adaptations to maternal exercise have yet to be determined. The origins and molecular contents, and, therefore, the resulting bioactivity is likely complex; nonetheless, our study provides a rationale for the continued exploration of exercise-induced small EVs in pregnancy to not only understand the potential impacts of small EVs on maternal-fetal health, but also the maternal physiological response to exercise. Further research is needed to clarify the kinetics and role of small EVs in the context of both acute and chronic exercise during pregnancy.

## Conclusions

We performed a novel examination of the exercise-induced small EV profile in pregnant women in comparison to non-pregnant controls. Although we found no differences in small EV size post-exercise, we found a significant increase in the concentration of small EVs in pregnant versus non-pregnant women after a matched-intensity treadmill exercise. The physiological relevance of small EVs in response to exercise in pregnancy warrants further examination. This study is a preliminary step to understand the role of small EVs in the physiological response to exercise in pregnancy.

## Supplementary Information

Supplementary Information.
