# Supplementary figures and images for: Circulating small extracellular vesicles increase after an acute bout of moderate-intensity exercise in pregnant compared to non-pregnant women
Source: Sci Rep. 2021 Jun 16;11:12615. doi: 10.1038/s41598-021-92180-5 (PMC8209031; doi:10.1038/s41598-021-92180-5)

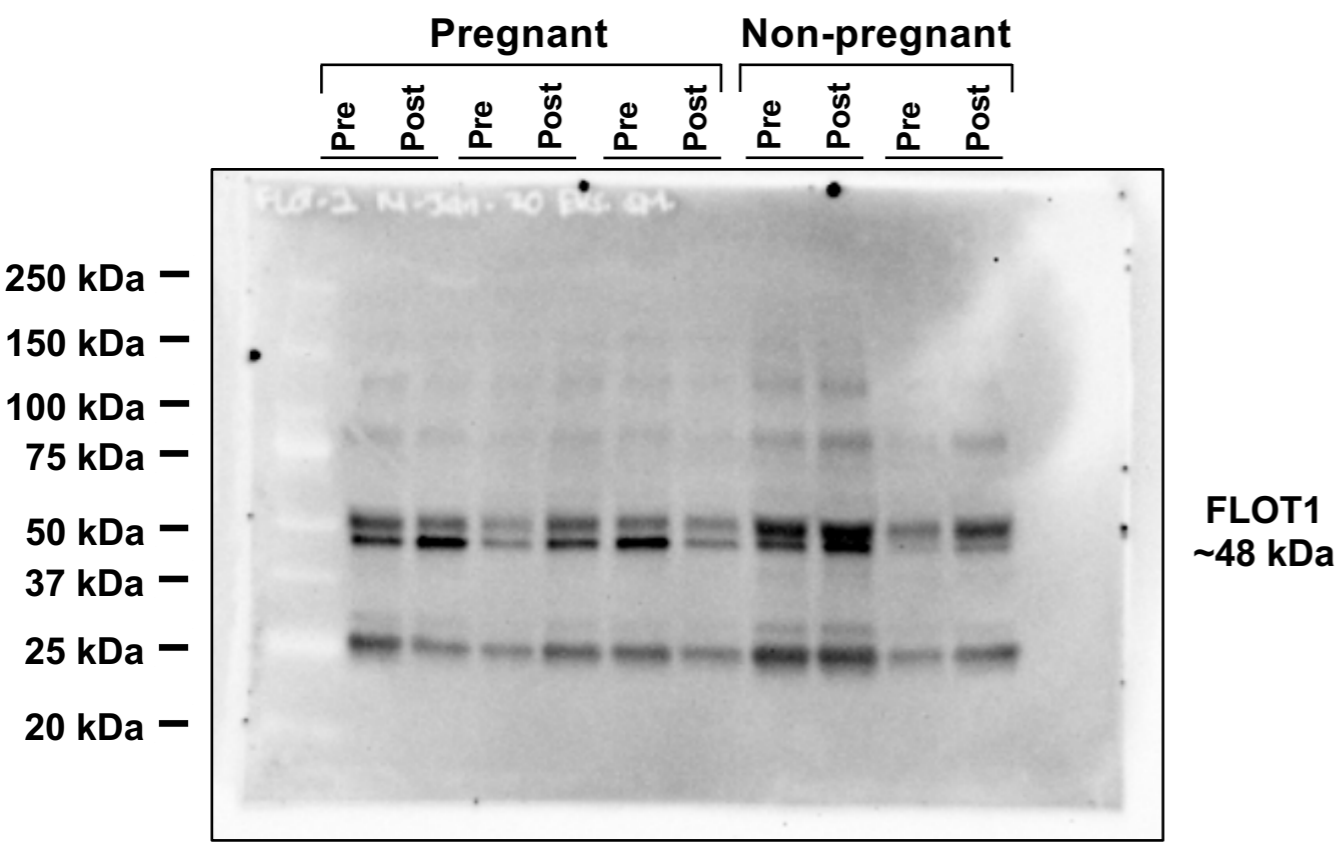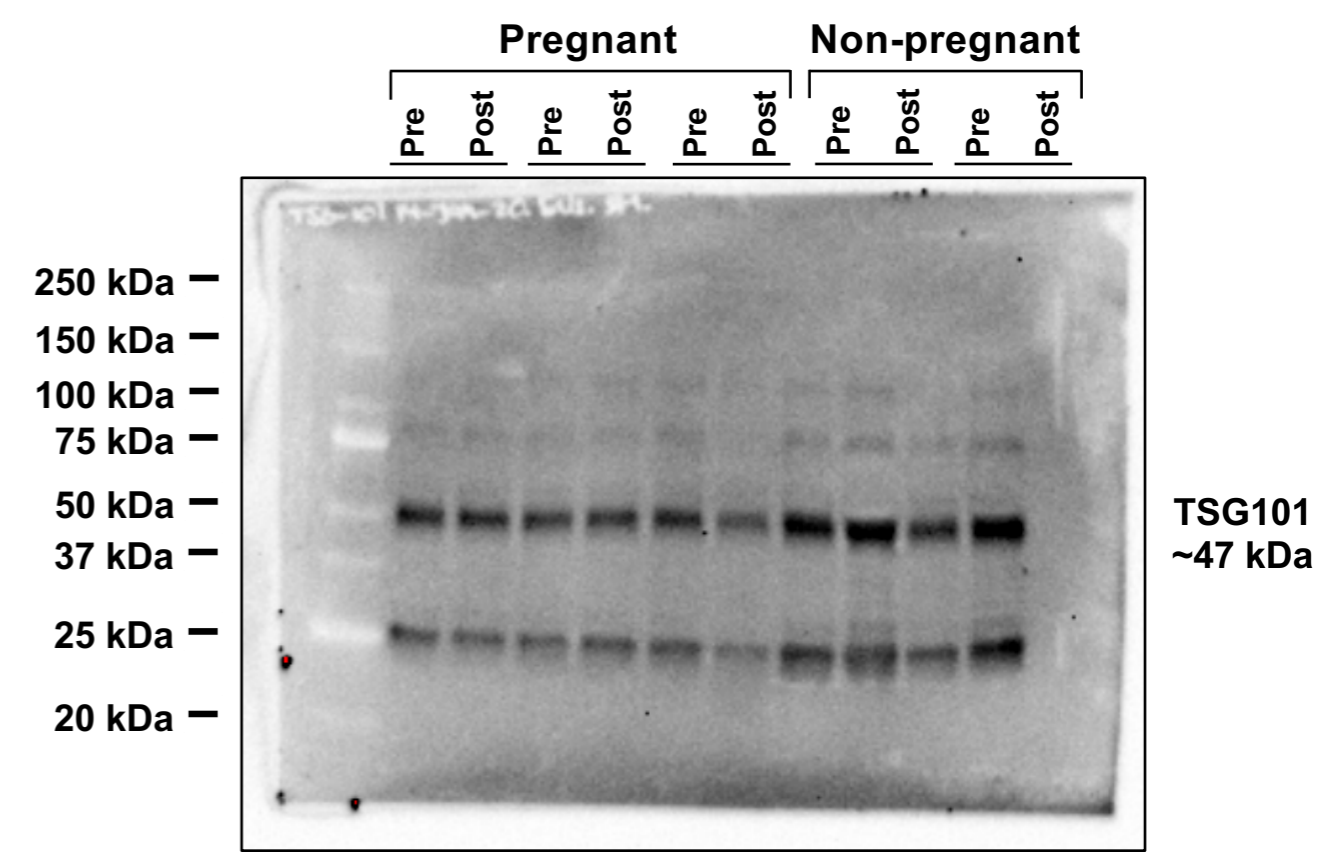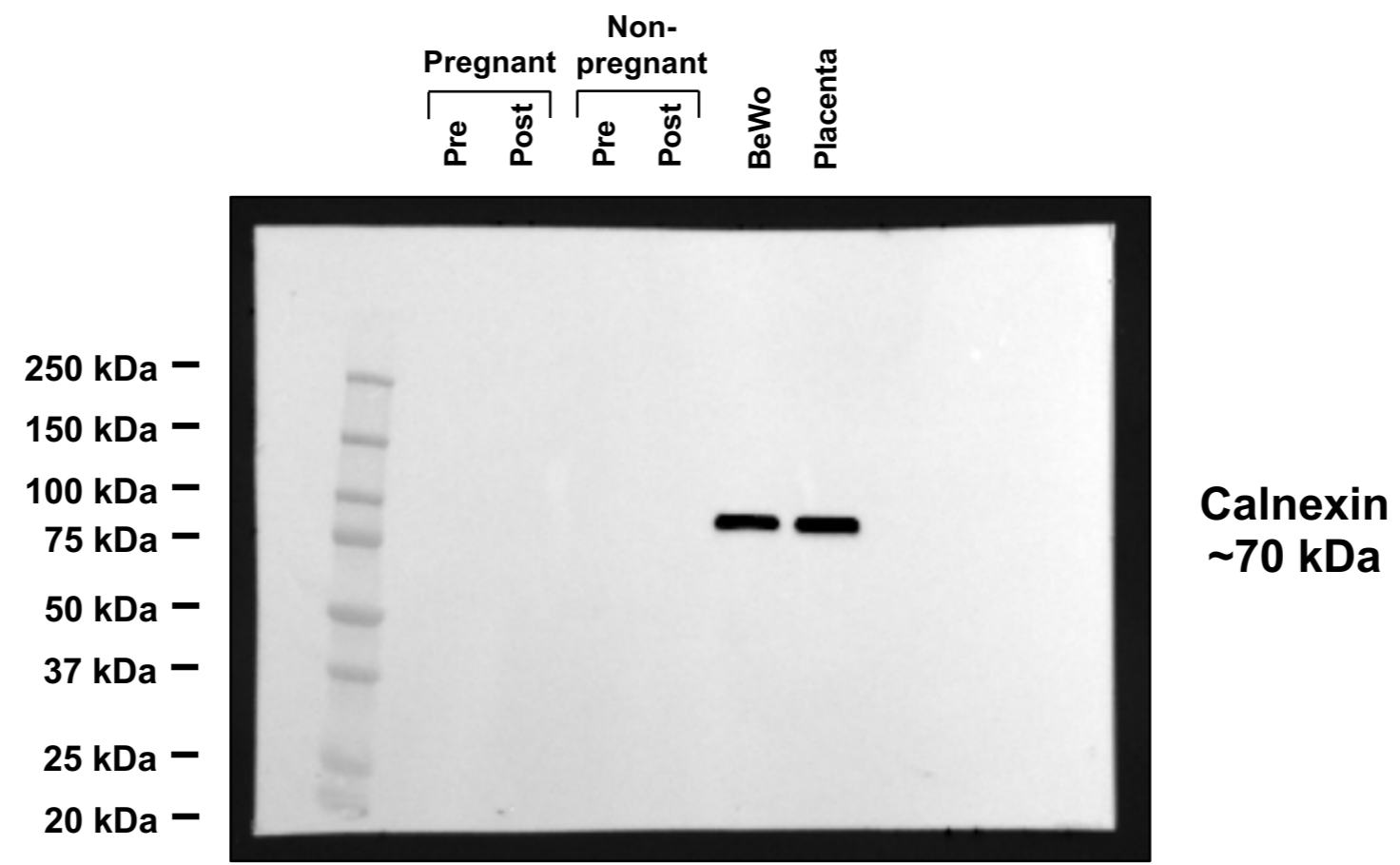

Supplement: Supplementary file 1 — Supplementary Information. [file 41598_2021_92180_MOESM1_ESM.pdf]
